# Tracheal reconstruction by re-inforced Gore-Tex in esophageal submuscular tunneling: An experimental study

**DOI:** 10.4103/1817-1737.74271

**Published:** 2011

**Authors:** Hossein Hodjati, Saeed Reza Baezzat, Afsoon Fazelzadeh, Nader Tanideh, Bita Geramizadeh

**Affiliations:** 1 *Department of Surgery, Shiraz University of Medical, Shiraz, Iran*; 2 *Trauma Research Center, Shiraz University of Medical Sciences, Shiraz, Iran*; 3 *Stem cell and Transgenic Technology Research Center, Shiraz University of Medical Sciences, Shiraz, Iran*; 4 *Department of Pathology, Shiraz University of Medical Sciences, Shiraz, Iran*

**Keywords:** Animal study, esophageal tunneling, tracheal reconstruction

## Abstract

**BACKGROUND:**

Tracheal reconstructions are aimed at rearranging or replacing parts of the tracheal tissue by different techniques. Here we introduce a new technique for tracheal reconstruction.

**METHODS:**

In 10 adult dogs, after intubation with an endotracheal tube, a segment of trachea including seven tracheal rings was resected circumferentially. A submuscular tunnel was induced between mucosal and muscular layers of the adjacent esophagus lying right next to the trachea. The esophageal submuscular tunnel starts and ends exactly at the level of distal and proximal ends of tracheal resection, respectively. Inforced Gore-Tex passed through the esophageal submuscular tunnel the distal segment of trachea and end-to-end anastomosis were made between distal ends of Gore-Tex and trachea, then endotracheal tube removed and the same procedure was made for proximal ends of Gore-Tex and trachea. Afterward, the proximal and distal ends of the esophageal tunnel were approximated to proximal and distal tracheal parts over the anastomosis.

**RESULTS:**

All dogs, except one due to anesthetic problem, survived and tolerated the operation; the first two dogs experienced postoperative fever, aspiration pneumonia, and died due to tracheoesophageal fistula. All survived animals were eating and barking well. We started to scarify dogs at least 6 and 12 weeks after operation for microscopy and pathologic examination. The Gore-Texes were patent and supported externally with fibrous connective tissue in esophageal tunneling, with in growth of respiratory epithelium on inner surfaces.

**CONCLUSION:**

Air tightness, good re-epithelialization, and relatively no limitation of esophageal length and no risk of luminal collapse are advantages of tracheal reconstruction by submuscular esophageal tunneling. This new method is worthy of further investigation, as it is technically feasible and easy to implement.

Experimental and clinical tracheal repair or anastomosis began in the late 19^th^ century. A few examples of limited tracheal resection and primary anastomosis were cited in the first half of the 20^th^century.[1]

Further experimental investigation on potential extent of tracheal resection and primary anastomosis without prosthesis greatly widened these possibilities. Approximately half of the adult trachea could be removed and primary reanastomosis performed[2–5] so, most tracheal lesions can now be resected and primary anastomosed safely.

The general limits of safe resection are about half of the tracheal length in adults and probably one third in small children. These patients are now treated with palliative techniques namely irradiation and stents or T tubes.

The reconstruction of the trachea after extensive resection remains a tremendous challenge and one of the most technically demanding of all thoracic procedures.

Numerous techniques have been described to date to confront the challenge of tracheal reconstruction.

The various tracheal substitutes and techniques of reconstruction were recently analyzed by Grillo,[1] who classified them in five categories: foreign materials (silicone tubes, coated stents, metallic, and other solid prostheses), nonviable tissues, autogenous tissues, tissue engineering, and tracheal transplantation. Attempts with foreign materials led to problems of chronic infection, airway obstruction, and migration of the prosthesis, erosion of major blood vessels, and proliferation of granulation tissue. Implantation of nonviable tissues, either chemically treated, frozen or lyophilized has been associated with poorly functional results. Reconstructions with autogenous tissues such as skin, fascia lata, pericardium, costal cartilage, bladder, esophagus or bowel are complex procedures, which have been associated with disappointing results. More recently, efforts have been made to induce the formation of cartilaginous tubes covered with epithelial cells, but to date this type of tissue engineering has not provided reliable results. Finally, tracheal allotransplantation has been also disappointing so far due to complication of necrosis or stenosis of the graft. In addition immunosuppressive therapy does not permit a clinical application in the treatment of cancer.[2–8]

In a previous work, we investigated living autologous aortic conduit to avoid immunologic reactions.[9] We found a progressive transformation of the vascular graft in to a structure resembling the tracheal tissue with mucociliary epithelium. This technique, however, had a limited value in clinical practice because of the need to remove an aortic segment from the patient himself with the risk of paraplegia. An aortic allograft would be a better practical solution, but potential problems of immunologic reaction could compromise the results. In addition, it was doubtful that the transformation observed with an autologous tissue would be reproducible with an allogenic tissue too.

In another study we introduced a new method of reconstruction of circumferential tracheal with submuscular esophageal tunneling.[10] In this method esophagus was not resected and there was no need for mucosal anastomosis but the issue of somewhat weak luminal support was an important concern in that study. In present study, to solve that problem we used an internal reinforced Gore-Tex prosthesis in submuscular esophageal tunneling, with specific characteristic such as structurally rigid enough to maintain an open lumen during unassisted ventilation’ biocompatible with smooth inner surface capable of supporting the growth of epithelium.

## Methods

This experiment was done on a total of 10 adult cross-breed dogs weighing 16.5 to 24 kg in animal research laboratory of Shiraz University of Medical Sciences. The procedures and the handling of the animals were reviewed and approved by the research and ethics committees of the Shiraz University of Medical Sciences (Shiraz, Iran) in accordance with the Principles of Laboratory Animal Care that have been formulated by the US National Society for Medical Research and with the Guide for the Care and Use of Laboratory Animals that has been published by the US National Institutes of Health (NIH publication 85-23, revised 1985, Washington, D.C.: US Department of Health and Human Services).

All procedures were carried out under aseptic conditions. The animals were kept in pathogen-free laboratory conditions (temperature, 20-25°C; humidified air; 12:12-h light-dark cycle). The protocols for anesthesia, operation, and sacrifice were identical for all animals. Anesthesia was induced by intravenous thiopental (15 mg/kg). Antibiotic therapy was done preoperatively using intravenous cefazolin sodium (100 mg/kg) on induction of anesthesia. In addition, intramuscular cefazolin sodium (100 mg/kg/d) was administered to dogs for a total of 7 days after the operation. After endotracheal intubation (using a cuffed endotracheal tube (ETT) no.8), the animals were maintained on controlled ventilation with halothane and 100% oxygen. The animal was prepared with povidone-iodine solution and draped; the skin and subcutaneous tissues were incised in the midline from 3 cm below the larynx to a length of 15 cm. The cervical trachea and esophagus were exposed by a midline separation of the paired strap muscles; the peritracheal sheath of the 10 tracheal rings was freed, and the trachea was mobilized circumferentially for 6 cm according to the planned resection.

**Figure 1:**
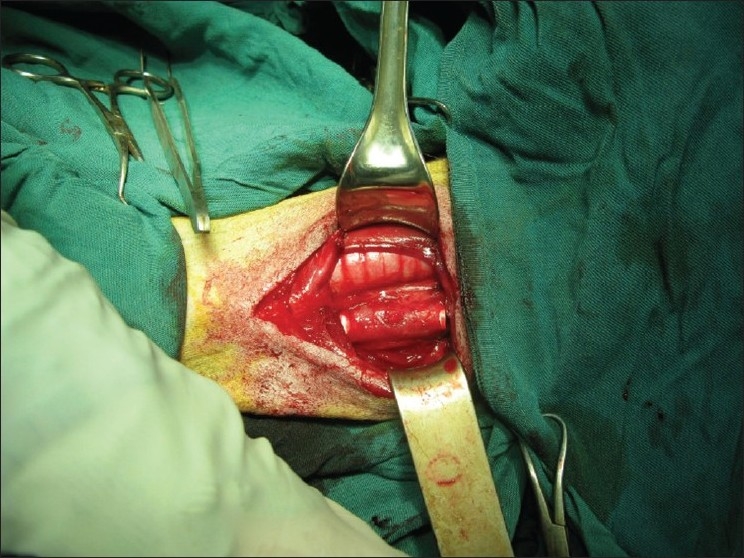
The esophageal submuscular tunneling with reinforced Gore-Tex

The adjacent esophagus lying right next to the trachea was mobilized gently to prevent vascular injury. Then about half the circumference of the anterior muscular part in both esophageal ends was incised and, by gentle and careful insertion of a tissue dissector between the mucosal and muscular layers of esophagus, a submuscular tunnel was made and about 8 cm reinforced gore-tex (Gore and associates. inc flag staff; Arizona 86004 USA Ako891-ml1) passed cautiously through the esophageal submuscular tunnel. A segment of trachea including seven tracheal rings (rings 5 through 11) was dissected and resected circumferentially. The animals were extubated and again re-intubated, using another cuffed ETT no. 7 via Gore-tex lumen and the distal segment of trachea and end to end anastomoses were made between distal ends of Gore-Tex and trachea over the ETT with a couple of running nonabsorbable 4-0 prolyn continuous sutures then ETT removed and the same procedure was made for proximal ends of Gore-tex and trachea. the esophageal submuscular tunnel starts and ends exactly at the level of distal and proximal ends of tracheal resection, respectively Afterward, the proximal and distal ends of the esophageal tunnel were approximated to proximal and distal tracheal parts over the anastomosis sites with running absorbable 3-0 Vicryl continuous sutures [Figure 1].

The strap muscles were re-approximated; the skin was closed in two layers with a couple of running absorbable 3-0 Vicryl sutures for subcutaneous tissue and interrupted 3-0 nylon sutures for skin, and then oxytetracycline antibiotic spray was applied.

The dogs were returned to their individual cages on the day of the operation. No oral feeding was given on the first and second postoperative days; fluid was resumed on the third day, and then the dogs were advanced to liquid and soft diet (without any hard bony component). The dogs were observed daily for coughing and dyspnea. The cervical incisions were evaluated daily for swelling, inflammation, seroma formation, and subcutaneous emphysema. Other cares were routine for the research facility.

In a preprogrammed manner, the surviving animals were put to death for macroscopic and microscopic observations by a rapid intravenous overdose of potassium chloride. Three dogs killed after 6 weeks and the 3 dogs were killed after 12 weeks postoperatively and were autopsied. One dog kept alive for long term result and sacrificed after 4 months at the end of study. The entire reconstructed trachea including the larynx and intra-esophageal parts was removed for histological evaluation. The anastomosis was evaluated for integrity, degree of luminal stenosis, granuloma formation, and histological response. After formalin fixation, specimens for histology were taken through the anastomosis, luminar surface of Gore-Tex and intra-esophageal parts, embedded in paraffin, stained with hematoxylin and eosin, and examined by means of light microscopy. All of the animals received humane care throughout the study.

## Results

All dogs, except one due to anesthetic problem, survived and tolerated the operation. The operation time was one hour on average. The first two dogs experienced postoperative fever, dyspnea with respiratory distress and died during the first and second post-operative week.

Postmortem examination revealed a small perforation of esophageal mucosa and formation of a 3-4 mm tracheoesophageal fistula. Otherwise all of the dogs were alive, eating and barking well and had uneventful post-operative courses until they were put to death.

**Figure 2:**
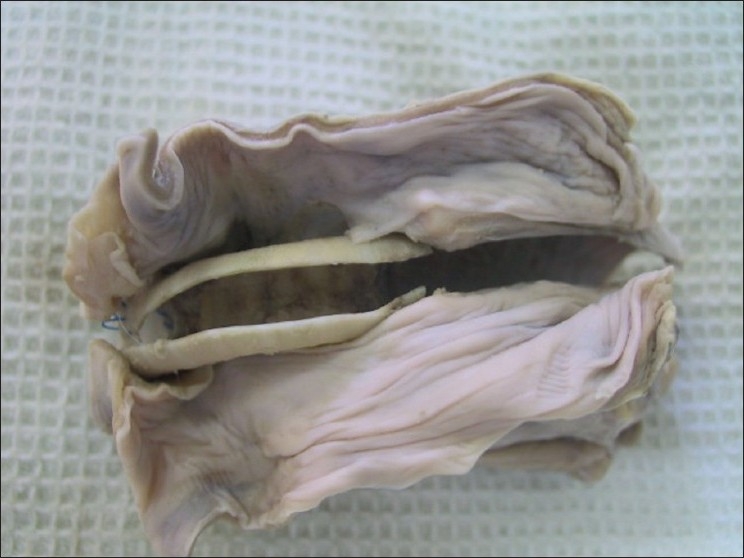
Necropsy; window-shape Gore-Tex graft on the submuscular tunneling in continuity with trachea

On post-mortem examination in neck exploration, there was no abscess or sign of infection in the site of graft. There was evidence of adhesion of adjacent muscles to the trachea. Both proximal and distal anastomosis had healed well, no significant anastomotic stenosis, sign of infection, any crust or deformity was found at the site of anastomosis. The Gore-Texes were supported externally with fibrous connective tissue in esophageal tunneling. Inner surfaces of Gore-Texes showed a well-defined area of gray with healed tissues [Figure 2].

Microscopic examination of hematoxylin and eosin stained of mucosa spanning the junction between the Gore-Tex and trachea demonstrated well-healed anastomosis in all dogs.

**Figure 3:**
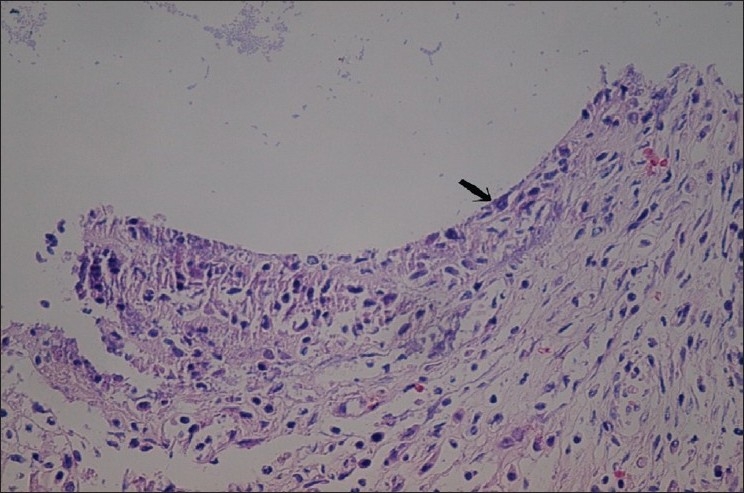
Photomicrograph of the longitudinal section taken from Gore-Tex graft 6 weeks after operation shows acute inflammation, fibrosis, and partial epithelialization with squamous metaplasia

In the section from Gore-Texes graft 6 weeks after operation showed that necrotic epithelium covered the entire surface, mild chronic inflammation, fibrosis, granulation and foci in growth of squamous metaplasia [Figure 3].

**Figure 4:**
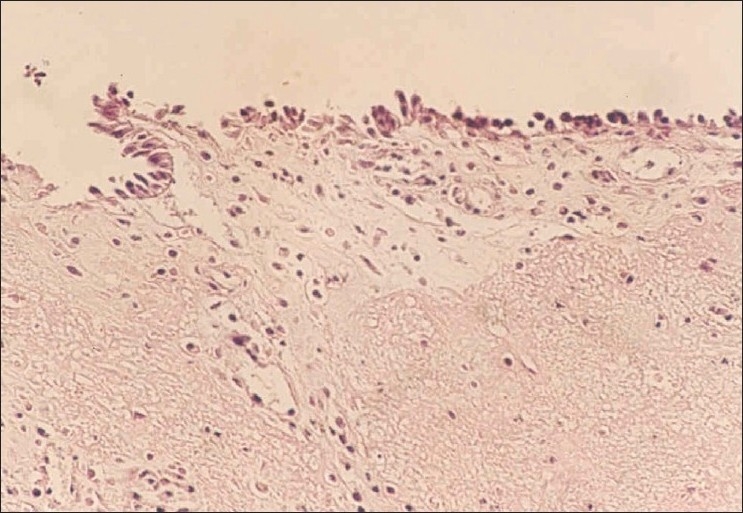
Photomicrograph of longitudinal section taken from Gore-Tex graft 12 weeks after operation shows mild chronic inflammation, fibrosis, and epithelialization with mixed squamous/mucociliary

In the section from Gore-Texes graft 12 weeks after operation showed chronic inflammation, fibrosis, and epithelialization with mixed squamous/mucociliary metaplasia [Figure 4].

## Discussion

Although most organs are successfully transplanted to date, the trachea remains an exception. Tracheal replacement with prosthetic or biological substitutes such as allografts or autologous grafts (trachea, esophagus, bowel, skin, or bladder) did not provide results satisfactory enough to lead to clinical application.[11–13]

Another line of experimental reconstruction with host tissue has been the use of adjacent esophagus to replace a long segment of trachea. Mural splinting is required to maintain patency, although, amazingly enough, in some experiments the splinting seems to have been unneeded. In one study, placement of a series of discontinuous polypropylene rings failed when the adjacent supported segments caused obstruction by sliding laterally. A suggestion that an esophageal segment be substituted for trachea just to provide a channel for a T tube[14] seems to overlook the magnitude of operation that is then needed for esophageal replacement. In an effort to avoid the necessity of a major operation for later esophageal replacement, revascularized segments of small intestine have also been used experimentally.[15] Given the collapsibility of small intestine, it is difficult to understand how respiration was possible, unless the interposed segment was very short.

Clinically, multi-staged cutaneous tubes have been widely used for construction of cervical trachea. Multiple trough techniques have been devised and used.[16,17] Edgerton and Zovickian[16] had success in multi-staged flap reconstruction of cervical trachea clinically. Grillo[18] described clinical application of his two-staged technique. Although successful in a few patients, the technique is not recommended because of an overall low success rate. It should be noted that staged repairs of the type listed have no application intrathoracically where an airtight airway must be attained immediately. Failure of healing, likely to occur in some cases after complex repairs, could cause fatal mediastinitis in the chest.

Esophagus *in situ* has also been used to patch the membranous tracheal wall and to serve as a long linear patch for congenital stenosis.[19,20] Fonkalsrud and colleagues[21,22] tried unsuccessfully to replace trachea with a segment of esophagus for agenesis and for stricture.

In a previous work, we reported that sub muscular esophageal tunneling could be a valuable substitute to the trachea for a period of 6 months.[10] But the issue of somewhat weak luminal support was an important concern in that study. To solve this problem our attention turned to meshes of various substances which might permit ingrowth of host connective tissue and this bed of autogenous new connective tissue would serve as a base for migration of adjacent tracheal epithelium. Also it can reinforce the luminal weakness. In the present study to solve that problem we used an internal reinforced Gore-Tex prosthesis in submuscular esophageal tunneling; with specific characteristic such as structurally rigid enough to maintain an open lumen during unassisted ventilation’ biocompatible with smooth inner surface capable of supporting the growth of epithelium.

In the present study, we replaced the cervical trachea with submuscular esophageal tunneling. Esophagus was not resected and there was no need for mucosal anastomosis. Therefore, there was low possibility of leakage and infection. We used this technique in reconstructing rather short tracheal defects. Due to formation of fibrous tissue between skeletal muscular structures of the neck and the external layer of the channelized esophagus, the new airway remained patent. We suppose that tracheal reconstruction by the esophageal tunneling technique has the potential to be considered reliable for the circumferential reconstruction of extensive laryngotracheal defects not amenable to being cured by conventional techniques.

In this method we do not need to keep ETT as a temporary stent and the Gore-Tex inhibits the collapse or stenosis of the tracheal lumen.

Yet, additional studies should be done on use of esophageal submuscular tunneling for reconstruction of larger tracheal defects, in other animals, or for the possibility of clinical application of this method. In addition, longer term studies should be done to evaluate the potential of granulation tissue formation and conduit stenosis in the new trachea.

## Conclusion

In conclusion, air tightness, good re-epithelialization, and relatively no limitation of esophageal length and no risk of luminal collapse are advantages of tracheal reconstruction by submuscular esophageal tunneling. This new method is worthy of further investigation, as it is technically feasible and easy to implement.
